# When context matters: community and environmental context to elicit natural products

**DOI:** 10.1042/EBC20250020

**Published:** 2026-08-19

**Authors:** Andres Cumsille, Josephine H.I. Putnam, Marc G. Chevrette

**Affiliations:** 1 Department of Plant Pathology, University of Wisconsin–Madison, Madison, WI 53706, U.S.A.; 2 Wisconsin Institute for Discovery, Madison, WI 53715, U.S.A.; 3 Microbiology Doctoral Training Program, University of Wisconsin–Madison, Madison, WI 53706, U.S.A.; 4 Department of Bacteriology, University of Wisconsin–Madison, Madison, WI 53706, U.S.A.

**Keywords:** microbial interactions, natural products, secondary metabolism

## Abstract

Natural products (NPs) comprise a wide range of bioactive compounds, including therapeutics and antibiotics. Despite several efforts, the discovery of novel NPs has been hindered by frequent rediscoveries of previously known NPs. Computational tools, such as those in genome mining, have helped identify the biosynthetic gene clusters (BGCs) responsible for the biosynthesis of NPs. However, there is a disconnect between the number of uncharacterized BGCs and the number of observed NPs in laboratory conditions. Considering the community and environmental context (C&EC) of NP-producing organisms can inform elicitation strategies to produce novel, uncharacterized NPs. Approaches inspired by nature, such as mimicking environmental conditions, introducing NPs as elicitors, and using multispecies co-cultures, have been proven successful in eliciting different responses in NP biosynthesis in the laboratory. However, the principles that drive how environmental conditions regulate NPs remain poorly understood. Integrating C&EC with genomic and metabolomic data enables the development of more rational, ecology-informed strategies for eliciting silent BGCs and discovering novel NPs.

## Introduction

Natural products (NPs) are the chemical products of living organisms, comprising a wide diversity of bioactive compounds that have been fundamental to the development of antibiotics and other therapeutics [1]. Discoveries of medicinally relevant NPs, such as antibiotics derived from microorganisms, highlight the importance of NPs, beginning with penicillin in 1928 and actinomycin, streptomycin, and more in the following decades [1,2]. These ushered in a ‘golden age’ of antibiotic discovery, uncovering new compound classes, many of which remain clinically important today. Despite increasingly sophisticated methods, modern NP discovery is challenged by frequent rediscoveries (*i.e.*, screens frequently yielding previously described NPs) [3,4].

Nevertheless, several advances have opened new ways to try to overcome this trend, especially by integrating ‘omics strategies through genome mining and/or metabolomics [5]. Mining genomes to identify biosynthetic gene clusters (BGCs) has enabled prioritizing NPs based on the pathways that assemble them and their presumed novelty, narrowing experimental focus toward novel secondary metabolites. Microorganisms produce a great diversity of NPs, whose natural relevance is often elusive and likely extends beyond the uses humanity has found for them (*e.g.*, human use of NPs as antibiotics, anticancer agents, immunosuppressives, and crop protectants) [6]. In nature, these compounds primarily function as mediators of survival and communication, *e.g.*, driving cellular processes and mediating intra- and inter-species interactions, influencing communication and survival across all environments [7].

An analysis of BGC diversity across bacteria by Gavriilidou et al. revealed that only ∼3% of bacterial BGCs have been experimentally characterized and linked to their chemical product [8]. Similar results have been predicted with nonribosomal peptides, where many novel BGC families have yet to be discovered, especially in under-sampled genera [9]. Despite extensive efforts, the range of NPs detected under standard laboratory conditions remains limited [4]. Bridging this divide between BGC diversity and observed NPs requires approaches that integrate genomic insight with ecological and community-informed perspectives. However, a major challenge of this approach lies in activating and expressing the estimated 80% of BGCs that remain silent under standard laboratory conditions [4,10,11].

Several efforts to elicit NP production have been made, including the ‘one strain many compounds’ (OSMAC) approach, which suggests that altering culture conditions (*e.g.*, media composition, culturing time, pH, and temperature) can increase a given strain’s repertoire of produced NPs [12]. Although OSMAC can boost the number of laboratory-observed metabolites in some cases, its success often relies on serendipity, and high rediscovery rates remain a critical challenge [2,13,14]. Another alternative to elicit production of silent NPs is the genetic engineering of BGCs, which can activate NP production and improve yields [15,16]. However, these techniques are often time-consuming, expensive, lineage-specific, and do not always yield consistent results.

Understanding microbial chemical interactions has prompted a conceptual shift from studying microorganisms in isolation toward recognizing them as members of complex, intertwined communities [17–20]. These natural interactions influence growth, gene expression, and NP production in ways that are difficult to comprehend, let alone reproduce under standard laboratory conditions. This untapped BGC diversity mirrors the ‘Great Plate Count Anomaly’ [21], which acknowledges a significant discrepancy between the diversity of direct microscopic counts of culturable bacteria and estimations using culture-independent techniques [22]: ∼99% of bacteria are ‘uncultivable’ under laboratory conditions [23–25]. Similarly, a minority of predicted BGC products can be observed under these same laboratory conditions. Our understanding of the stimuli and mechanisms that regulate BGCs and control NP biosynthesis is incomplete, often disconnected from nature, and limited to anecdotal examples [7]. Both the ‘Great Plate Count’ and the BGC-product anomalies suggest the important context of their natural environments, where community dynamics and interspecies interactions allow for microbes to live, thrive, and respond to each other.

**Figure 1:**
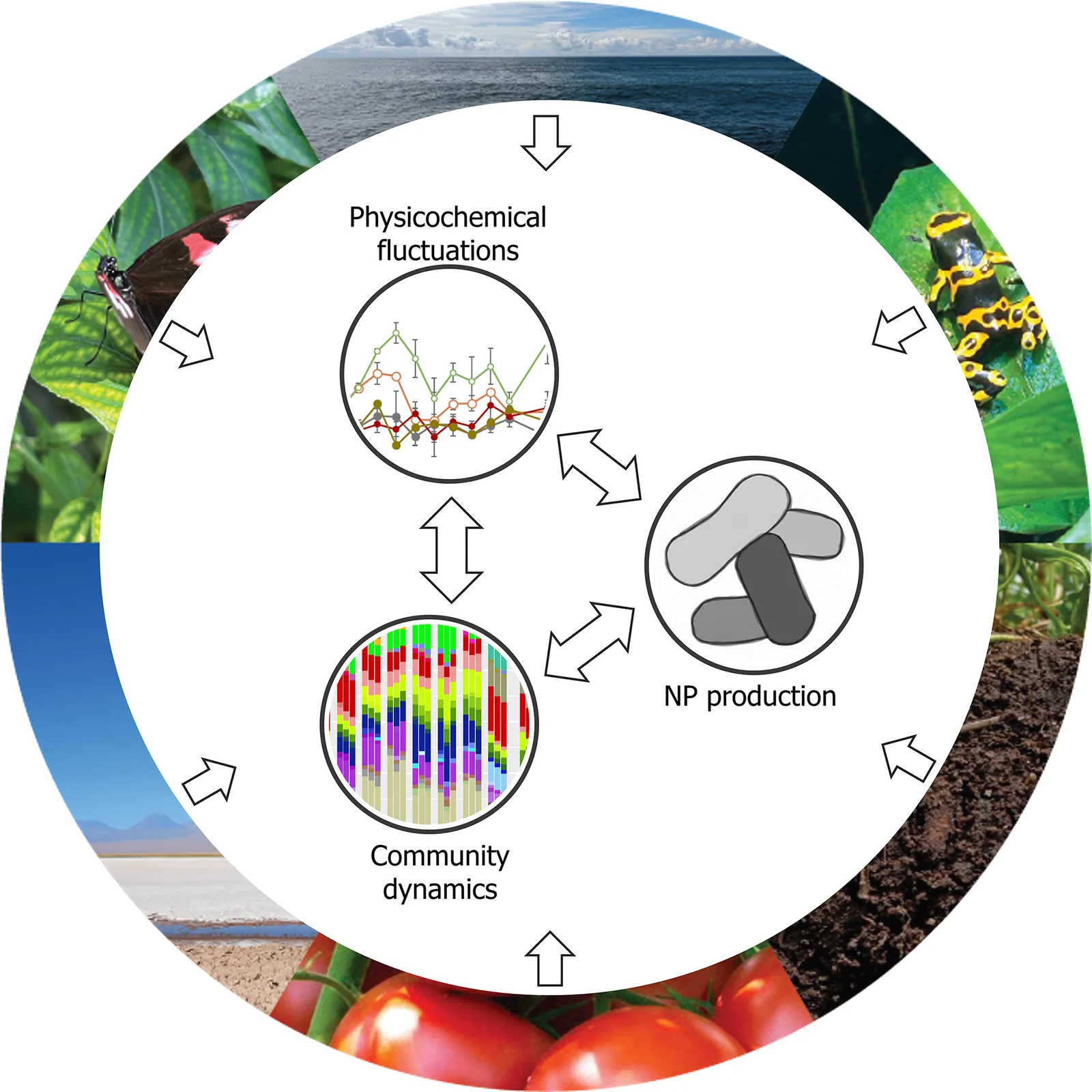
Factors altering the production of NPs Physicochemical fluctuations, community dynamics, and NP production are highly interconnected and are all strongly influenced by the environment (outer ring in the figure).

Microorganisms dwell in nutrient-diverse ecosystems, which comprise several spatial and temporal niches where multiple species compete and cooperate [11,26]. Physicochemical changes (*e.g.*, in pH, humidity, oxygen, iron, salts, temperature, UV exposure, etc.) reshape fitness landscapes and introduce varying selective pressures, altering community composition, which in turn affects NP production of individuals. Physicochemical fluctuations, community dynamics, and NP production are tightly interconnected, each influencing and being influenced by the others, and ultimately being influenced by the environment (Figure 1).

In the environment, microorganisms typically grow at rates 50–100× slower than in laboratory conditions [6], and ∼60% of global microbial biomass is in a nongrowing state [27]. When we take microorganisms from their environment and study them as monocultures in the lab, they often exhibit different growth strategies and gene expression dynamics. Given the contextuality of microbial behavior, it is expected that microorganisms produce certain NPs only within particular consortia [20]. Moreover, different niches can shape (and reshape) microbial communities through time and space; even transient states before reaching equilibrium can create ideal conditions to produce an NP in certain communities.

Microorganisms talk to each other [6], and we do not yet understand what they are saying or to whom they are speaking. Elucidating these interactions *in situ* remains a major challenge for microbiologists. Considering community and environmental context (C&EC) may provide deeper insight into the triggers of NP biosynthesis and may inform strategies to produce uncharacterized NPs and suggest their ecological roles. From a practical perspective, embracing C&EC means developing approaches that bring laboratory conditions closer to those found in nature. Key opportunities lie in exploring how abiotic factors, chemical signaling, and microbial interactions can shape NP production. The following sections examine C&EC in greater detail: first, environmental and media variations as elicitors in culture conditions and how recreating the physicochemical conditions of the isolation niche might stimulate NP biosynthesis; second, the role of NPs as potential elicitors; and third, how incorporating multispecies community co-cultures can influence NP discovery and production.

## Environmental and media variations as elicitors in laboratory cultures

OSMAC has been widely applied to NP discovery; however, similar strategies have long been used to isolate bacteria from diverse ecosystems. Isolation media are often designed to incorporate elements from the source environment, thereby mimicking conditions that support bacterial survival in their respective ecosystems. Examples include humic acid-vitamin agar (resembling soil [28]), chitin medium (commonly used for insect-associated bacteria [29]), and media to mimic marine environments (*e.g.*, actual or artificial seawater [30], or adding sea sponge [31] or sea urchin [32] extracts).

A notable technology to isolate novel taxa and discover NPs is the ‘iChip,’ which enables high-throughput, *in situ* cultivation by growing single, environmentally isolated cells in separate diffusion chambers that exchange nutrients and signals with their native habitats. The iChip strategy substantially increases access to novel taxa and metabolites [33], exemplified by the discovery of the novel antibiotic teixobactin from a previously uncultured bacterium [34].

Although widespread in microbial isolation studies, these strategies that mimic the source environment are less common in NP elicitation. This may in part be due to the difficulty of replicability and scale-up of culture conditions. However, these efforts could be valuable to understand how environmental stimuli impact NP production [35]. Varying carbon sources and other media components show promise in affecting if bacteria induce or repress NP production. An example is the β-lactam cephamycin C produced by *Streptomyces clavurigerus*, whose production is suppressed in the presence of glycerol [36,37]. Actinorhodin, a polyketide produced by *Streptomyces* spp., is down-regulated in the presence of glucose [37]. Other examples have been observed in *Streptomyces coelicolor*. Exposure to cold, heat, ethanol, salt, or a protein synthesis inhibitor resulted in different proteomes, suggesting differential stress regulatory responses [38]. Likewise, cold shock can interfere with translation and trigger stress responses. Such is the case in *Chromobacterium subtsugae* (formerly *C. violaceum*), which produces violacein at 16°C, highlighting that cold shock can influence NP biosynthesis [39].

The high-throughput elicitor screening platform is an effort to systemize the use of abiotic elicitors for NP discovery. First demonstrated in *Burkholderia thailandensis*, a large collection of bioactive compounds can lead to the production of NPs and virulence factors that are not normally produced without an inducer [10]. In actinomycetes, chemically diverse abiotic elicitors (*e.g.*, flavonoids, clinical drugs, and other small molecules) can induce the production of antibiotic NPs with activity against both Gram-positive and Gram-negative bacteria [40].

While several studies demonstrate that modifying culture conditions can successfully elicit novel NPs, the underlying principles that govern how environmental conditions regulate secondary metabolism are still poorly understood. NP discovery often depends on the serendipity of lucking into the right conditions.

## Incorporating natural products into laboratory cultures

While antibiotics have long been understood as weapons that bacteria can use to weaken or kill their competitors, most antibiotics likely do not reach lethal concentrations in natural environments [41], suggesting antibiotics may serve other natural functions. Roles such as coordinating mutually beneficial activities, like biofilm formation, and manipulating other members of their microbial community have been proposed [42]. In addition, antibiotic resistance genes are found in every ecosystem with microbial communities present and are common in a wide range of bacterial species, suggesting that antibiotic resistance has existed for as long as antibiotics [43].

Sublethal concentrations of antibiotics can induce gene expression changes and NP production. For example, in *Salmonella typhimurium*, treatment with a variety of sublethal antibiotics causes a global transcriptional response [44]. In *B. thailandensis*, antibiotics lethal at high concentrations act as inducers of silent BGCs at lower concentrations [10,45]. Using this approach, Xu et al. demonstrated that NPs could be used to activate a silent gene cluster in *Streptomyces albidoflavus* (formerly *S. albus*) [46], namely etoposide and ivermectin, enabling the isolation and structural characterization of 14 previously undescribed NPs.

Following these findings, the same group [40] found that several NPs can induce antibacterial activity against *Bacillus subtilis* in various Actinobacteria. Diverse compounds, ranging from flavones to clinical drugs, elicited the production of antibacterial NPs [40]. Similarly, Lozano et al. [39] induced production of the antibiotic violacein in *C. subtsugae* with sublethal antibiotic treatment. Global RNA sequencing analysis revealed that tested antibiotics up-regulated the expression of genes involved in NP biosynthesis, transport, and catabolism, ultimately shaping *C. subtsugae*’s chemical output.

These findings support the role of NPs as signal molecules and the utility of their sublethal administration in eliciting the production of other NPs. In nature, antibiotic NPs are most likely produced at low sublethal concentrations, serving other functions (*e.g.*, as signals [6,43]). NPs likely trigger responses within microbial communities, where molecules from one organism act as cues for other organisms to produce other molecules. In this framework, NPs are produced and exchanged as part of dynamic and interconnected chemical communication networks. This suggests that using NPs and/or sublethal concentrations of antibiotics as an elicitation strategy in the laboratory may reflect processes that occur naturally within microbial communities. In this context, antibiotic production and sensing are integral components of community-level interactions rather than isolated, unidirectional responses.

## Incorporating community dynamics into the laboratory

Another emerging approach that has proven successful is incorporating multispecies co-cultures and community dynamics into laboratory conditions, rather than studying microorganisms in isolation, to elicit shifts in NPs’ production.

Ueda et al. analyzed pairwise interactions among 76 *Streptomyces* strains and observed many responses (*e.g.*, antibiotic production, aerial mycelium formation, and sporulation) that they attributed to diffusible substances. The authors suggest that bacteria in nature may engage in cooperative interactions where NPs act as molecular signals that can in turn trigger the production of other NPs and affect life cycle dynamics. Such interactions may be even more common in natural ecosystems than we appreciate [47].

Multispecies co-cultures of bacteria have resulted in the identification of several compounds not produced in monoculture. *Streptomyces endus* co-cultured with *Tsukamurella pulmonis* produced alchivemycin A, a novel antibiotic with activity against *Micrococcus luteus* [48]. Similarly, co-culturing marine-derived *Micromonosporaceae* with *Rhodococcus* identified 12 *Micromonospora* spp. that produce unique metabolites, many with antibacterial activity [18]. Following that study, the antibiotic keyicin was discovered when coculturing *Rhodococcus* with *Micromonospora* [49], where keyicin production was induced by supernatants of *Rhodococcus* and other mycolic acid-containing bacteria [50].

Other examples of NP-induced elicitation include *Streptomyces lividans*, which triggers a spreading population phenotype in *B. subtilis* when co-cultured. *B. subtilis*, in turn, stimulates prodiginine production in *S. lividans* [51]. Similarly, *S. coelicolor* has displayed differential NP production in response to several bacterial interactions (*e.g.*, *Amycolaptosis* and other actinomycetes elicit the production of actinorhodin, prodiginine, desferrioxamines, and coelichelin [52]). Likewise, co-culture with *Myxococcus xanthus* elicits actinorhodin in *S. coelicolor* [53].

Differential NP production upon co-culture has not only been observed in actinomycetes. For instance, *C. subtsugae* produces the NP violacein in response to hygromycin production by *Streptomyces* sp. 2AW. This response was further proven to be caused by any antibiotic that inhibits the peptide elongation during translation [39]. Violacein production in *C. subtsugae* is regulated downstream of quorum sensing, suggesting that this signal cascade itself may respond to or influence antibiotic cues [20]. Such regulation implies potential quorum-sensing crosstalk among interacting species, further emphasizing the complexity of these co-culture dynamics. Lozano et al. further questioned whether the induction of pigmented NPs, such as violacein, might be a broader elicitation phenomenon that is not frequently observed in non-pigmented metabolites [39].

Other model bacterial communities add complexity beyond pairwise interactions, such as the hitchhikers of the rhizosphere (THOR) community. Here, *Pseudomonas koreensis* produces koreenceine, which inhibits the growth of *Flavobacterium johnsoniae*. However, when *Bacillus cereus* is incorporated into three-member cocultures, it rescues *F. johnsoniae* from this inhibition [19]. THOR BGC dynamics have been explored in depth in monocultures, pairwise co-cultures, and the full three-member community, displaying differential BGC expression, especially in *B. cereus* in the presence of *P. koreensis* (*e.g.*, repression of siderophore production when koreenceine is present [54,55]). In addition, different analogs of lokisin, produced by *P. koreensis*, were observed to be dependent on co-culture member composition, suggesting molecular hand-offs between species [54].

Another three-member bacterial community demonstrates the complexity of microbial interactions. This community consists of *Pseudomonas syringae, B. thailandensis*, and *C. subtsugae*. In this community, B. *thailandensis* inhibits *P. syringae*, and differential metabolite production is observed in the three-member community, dependent on the growth phase [20] and community composition/complexity [27].

Weiss et al. studied the mouse microbiota [56] through a 12-member synthetic bacterial community. The authors used pairwise interactions of spent media and evaluated growth and metabolic interactions, observing that most interactions were amensalistic or competitive and that one of the members, *Enterococcus faecalis* KB1, which plays a dominant role in pairwise interactions, produces antibacterial compounds only in a subset of these conditions [57].

Each bacterial interaction study raises new questions. For example, in what conditions and communities are particular NPs produced? How might more complex community dynamics influence these interactions? What elicitor molecules trigger the production of others? While adding complexity to synthetic bacterial communities is challenging, the question remains whether these lab-observed phenotypes can truly replicate those found in nature. Moving beyond pairwise interactions toward more complex microbial communities may provide a more realistic framework to study NP production. Evaluating how interspecies interactions shape community dynamics, particularly when coupled with metabolomic analyses, can provide direct insights into how specific interactions influence the production of NPs. At the same time, incorporating detailed information on nutrient availability and media composition can help elucidate how biotic and abiotic factors work in concert to regulate these communities.

## Moving forward

Despite the rapid expansion of genome mining and bioinformatic tools, most BGCs remain silent under standard laboratory conditions, and we are unable to observe/detect their products. Many studies that aim to closely mimic natural conditions using abiotic elicitation, sublethal antibiotic exposure, or microbial co-cultures [58] have demonstrated that NP biosynthesis is highly context-dependent. In fact, several studies have sought to standardize host-microbiome interactions, including the reproducible fabricated microbial ecosystems. This approach holds promise for NP discovery by mimicking micro-ecosystems and enabling the study of microbial communities under controlled conditions [59].

To move beyond this trend in NP discovery, the field may need to rethink how new NPs are detected. In particular, we need to consider both the dark metabolome (the entirety of molecules an organism can produce in their environmental context) and the dark matter of metabolomics (all detected, but unannotated signals in a metabolomics data set) [60]. Addressing these gaps will require us to acknowledge the limitations of current methodologies, develop new approaches, and generate more biological data to expand public datasets and therefore improve our understanding of the roles that NPs play across different contexts [61]. Progress will also depend on community effort, including open-access data sharing and improved annotation of metabolomics datasets. One example is the Collaborative Microbial Metabolite Center Knowledgebase (CCMC-KB), which aims to collect and curate knowledge about microbial metabolites from metabolomic datasets [62].

A key gap in the field lies in the dark metabolome and our limited understanding of how environmental conditions and interspecies interactions converge to regulate NP production. There is a need to shift from random NP discovery pipelines toward rational, integrative frameworks that incorporate microbial ecology. Combining metabolomics with community-level approaches, such as 16S rRNA metabarcoding and shotgun metagenomics, can provide temporal (and sometimes functional) insights into when and under what conditions specific metabolites are produced. Similarly, long-read co-assembled metagenomes paired with metatranscriptomes have been used to uncover the secondary metabolic potential of uncultivated microbes and to explore community responses to environmental changes [63].

Co-occurrence networks have previously been used to infer the dynamics of uncultured bacteria [64–66]. These networks could inform the rational design of cultivation and elicitation strategies that mimic more C&EC in natural ecosystems. Co-occurrence networks offer a promising avenue to generate testable hypotheses about microbial interactions and the environmental cues that drive NP biosynthesis. These networks provide a framework to evaluate potential interactions among microorganisms, potentially revealing interactions that drive co-occurrence or exclusion within a given environment [65–67].

When coupled with metabolomic data and other physicochemical information from the same ecosystems, co-occurrence networks might deepen our understanding of the biological and chemical contexts required to isolate novel taxa and/or induce the production of new NPs. Chase et al. integrated untargeted metabolomics data from marine sediments with shotgun metagenomic and 16S rRNA sequencing. The study revealed that biosynthetic potential, community composition, and metabolomic profiles are largely structured by local biogeographic patterns, with metabolomes showing strong site-specific signatures. Overall, the findings suggest that C&EC plays a major role in shaping biosynthetic and metabolic diversity in marine sediments [68].

NP biosynthesis is shaped by ecological context and microbial interactions that are largely missing in traditional laboratory setups. Integrating C&EC, including ecological context, community analyses, and metabolomics, into NP discovery pipelines represents a critical step toward unlocking silent biosynthetic potential and understanding the biological roles of these metabolites in nature. Not only will this give us basic insight into the fundamental processes that govern microbial chemical interactions, but it may also serve as a blueprint for the more applied aspects of NP science, including where to efficiently find new therapeutics and how to upscale their production.

## Summary

- There is a great divide between predicted BGCs and observed NPs in the laboratory.
- Considering C&EC may reveal how NPs are elicited and guide strategies for the discovery of novel, uncharacterized NPs.
- Mimicking nature, through media adjustment, adding NPs as elicitors, or multispecies co-cultures are strategies that could help elicit NP production and understand the ecological role of NPs.

## Open Access

Open access for this article was enabled by the participation of the University of Wisconsin - Madison in an all-inclusive Read & Publish agreement with Portland Press and the Biochemical Society under an agreement with the Big Ten Alliance.

## Abbreviations

BGC: biosynthetic gene clusters
C&EC: community and environmental context
NPs: natural products
OSMAC: one strain many compounds
THOR: the hitchhikers of the rhizosphere
